# Potential for airborne transmission of infection in the waiting areas of healthcare premises: stochastic analysis using a Monte Carlo model

**DOI:** 10.1186/1471-2334-10-247

**Published:** 2010-08-20

**Authors:** Clive B Beggs, Simon J Shepherd, Kevin G Kerr

**Affiliations:** 1 Bradford Infection Group, School of Engineering, Design and Technology, University of Bradford, Bradford, BD7 1DP, UK; 2 Department of Microbiology, Harrogate and District NHS Foundation Trust, Harrogate District Hospital, Lancaster Park Road, Harrogate, HG2 7SX, UK

## Abstract

**Background:**

Although many infections that are transmissible from person to person are acquired through direct contact between individuals, a minority, notably pulmonary tuberculosis (TB), measles and influenza are known to be spread by the airborne route. Airborne infections pose a particular threat to susceptible individuals whenever they are placed together with the index case in confined spaces. With this in mind, waiting areas of healthcare facilities present a particular challenge, since large numbers of people, some of whom may have underlying conditions which predispose them to infection, congregate in such spaces and can be exposed to an individual who may be shedding potentially pathogenic microorganisms. It is therefore important to understand the risks posed by infectious individuals in waiting areas, so that interventions can be developed to minimise the spread of airborne infections.

**Method:**

A stochastic Monte Carlo model was constructed to analyse the transmission of airborne infection in a hypothetical 132 m^3 ^hospital waiting area in which occupancy levels, waiting times and ventilation rate can all be varied. In the model the Gammaitoni-Nucci equation was utilized to predict probability of susceptible individuals becoming infected. The model was used to assess the risk of transmission of three infectious diseases, TB, influenza and measles. In order to allow for stochasticity a random number generator was applied to the variables in the model and a total of 10000 individual simulations were undertaken. The mean quanta production rates used in the study were 12.7, 100 and 570 per hour for TB, influenza and measles, respectively.

**Results:**

The results of the study revealed the mean probability of acquiring a TB infection during a 30-minute stay in the waiting area to be negligible (i.e. 0.0034), while that for influenza was an order of magnitude higher at 0.0262. By comparison the mean probability of acquiring a measles infection during the same period was 0.1349. If the duration of the stay was increased to 60 minutes then these values increased to 0.0087, 0.0662 and 0.3094, respectively.

**Conclusion:**

Under normal circumstances the risk of acquiring a TB infection during a visit to a hospital waiting area is minimal. Likewise the risks associated with the transmission of influenza, although an order of magnitude greater than those for TB, are relatively small. By comparison, the risks associated with measles are high. While the installation of air disinfection may be beneficial, when seeking to prevent the transmission of airborne viral infection it is important to first minimize waiting times and the number of susceptible individuals present before turning to expensive technological solutions.

## Background

Although many infections that are transmissible from person to person are acquired through direct contact between individuals [1], a minority, notably tuberculosis (TB) [2-5], measles [6] and influenza [7], are known to be spread by the airborne route. Airborne infections pose a particular threat to susceptible individuals whenever they are placed together with the index case in confined spaces [5]. Indeed, numerous outbreaks have occurred due to the liberation of airborne infectious particles in enclosed spaces, some involving large numbers of people [8-11]. With this in mind, waiting areas of healthcare facilities present a particular challenge [12], since large numbers of people, some of whom may have underlying conditions which predispose them to infection, congregate in such spaces and can be exposed to an individual who may be shedding potentially pathogenic microorganisms. It is therefore important to understand the risks posed by infectious individuals in waiting areas, so that interventions can be developed to minimise the spread of airborne infection.

While much has been written concerning the spread of airborne infection in buildings, most of this work has focused on individuals who spend days, or even weeks, in an enclosed space [8,13,14]. By comparison, very little work has been undertaken on those applications, such as waiting areas, where individuals spend comparatively little time in an enclosed space. When exposure times are short, the risk that any transmission will occur is strongly influenced by chance events. Consequently, when evaluating risk, deterministic methodologies are only of limited value. Stochastic methodologies are much more appropriate, as these allow the effect of chance variations to be readily evaluated. Therefore, in order to gain a greater understanding of the risks associated with airborne transmission when exposure times are short, we constructed a stochastic Monte Carlo model using the Gammaitoni-Nucci equation [5,15] to calculate the risk posed by infected individuals in the waiting areas of healthcare facilities.

## Method

A stochastic Monte Carlo model was constructed, using Microsoft Excel, to analyse the transmission of airborne infection in waiting areas. In this model we utilized the Gammaitoni-Nucci equation [15] below (equation 1), to predict probability of susceptible individuals becoming infected.

(1) $$P=1-e^{\left[-\frac{pI\phi }{V}\left(\frac{Nt+e^{-Nt}-1}{N^{2}}\right)\right]}$$

Where *P *is the probability of infection of susceptible individuals, *p *is the pulmonary ventilation rate (m^3^/h), *I *is the number of infectors, *ϕ *is the quantum generation rate (quanta/h), *V *is the room volume (m^3^), *N *is the room ventilation rate (air changes/h), and *t *is the exposure time for susceptible individuals (h).

Equation 1 is derived from the following fundamental equations for the rate of change of susceptible individuals and quanta with time:

(2) $$\frac{dS}{dt}=-\frac{p}{V}nS$$

(3) $$\frac{dn}{dt}=-Nn+q$$

Where, *S *is the number of susceptible individuals, *t *is time (h), *n *is the number of quanta of infection in the air (quanta), and, *q *= *Iϕ*, i.e. the total rate of quanta generation by all infectors (quanta/min).

Gammaitoni and Nucci solved these equations to give the quanta in the air at time *t, n*_*t*_, as:

(4) $$n_{t}=\frac{q}{N}+\left[n_{0}-\frac{q}{N}\right]e^{-Nt}$$

and the number of susceptible persons at time *t*, for an initial quanta level of *n*_0 _= 0 as:

(5) $$S_{t}=S_{0}e^{\left[-\frac{pq}{V}\left(\frac{Nt+e^{-Nt}-1}{N^{2}}\right)\right]}$$

Where, *S*_*t *_is the number of susceptible individuals at time *t*, and *S*_0 _is the number of susceptible individuals at time *t *= 0 h.

Mathematical models examining the spread of airborne infection in confined spaces were first developed by Wells [16]. In an attempt to describe the stochastic behaviour of airborne infection, he introduced a unit of infection termed the 'quantum', defined as the infectious dose required to infect (1 - e^-1^) (i.e. 63.2%) of the people in an enclosed space. Despite its stochastic definition, the number of quanta in a room is generally considered to be a physical measure of the infectious material present, which effectively indicates both the quantity and pathogenicity of the infectious material present in the air, as well as the average susceptibility of the individuals in the enclosed space. Riley *et al*. [6] modified Wells' original model, to give an expression known as the Wells-Riley equation [5,8], reflecting the exponential increase in the number of new cases of infection with time for steady-state quanta levels in a room space. In our study, because we were interested in transmission that might occur during the relatively short periods of time in which patients spend in waiting rooms, we chose to the use Gammaitoni-Nucci equation (which is identical to that described by Rudnick and Milton [17]) - a modified version of the Wells-Riley equation, that takes into account transient behaviour over short periods of time. According to the Gammaitoni-Nucci equation, the number of new infections, *C*, is given by:

(6) $$C=S\left(1-e^{\left[-\frac{pI\phi }{V}\left(\frac{Nt+e^{-Nt}-1}{N^{2}}\right)\right]}\right)$$

Where S is the number of susceptible individuals present.

In our model it was assumed that:

• Only one infectious individual is present in the waiting room during each simulation.

• While susceptible individuals may become infected, they cannot infect anyone else in the waiting room because they leave it before they themselves become infectious.

• The waiting room is ventilated with outside air at a constant flow rate.

• The air in the waiting room is well mixed, so that infectious particles are evenly distributed throughout the room space.

• The values of *p*, *ϕ*, *t *and *S *vary and are normally distributed.

In order to allow for the inherent stochasticity associated with airborne infection, a total of 10000 individual simulations were undertaken. In each simulation the Gammaitoni-Nucci equation was used to calculate the risk of susceptible individuals becoming infected, assuming that one infectious person was present in a waiting room at all times. While mean values and standard deviations for *p*, *ϕ*, *t *and *S *were specified by the user, to ensure stochasticity a normally distributed random number generator was used in the model to determine the precise values of these variables for each simulation.

We used our model to evaluate the spread of the three real and one hypothetical infection characterized in Table 1. The quanta production rates for the influenza and measles outbreaks were values used by Rudnick and Milton [17], while the value for TB was that derived by Nardell *et al*. [8] from an outbreak in the USA. These values were chosen because they are indicative of the relative infectivity of the various diseases. For illustrative purposes we added a hypothetical infection with an airborne route of transmission in which the quanta production rate was very high. For each of these infections we applied a standard deviation equal to 25% of the mean quanta production value. We applied the data in Table 1 to a default waiting room characterized by the assumed data in Table 2.

**Table 1:** Characteristics of the airborne infections analysed in the study

Infectious agent	Mean quanta production rate (quanta/h)	Standard deviation (quanta/h)
*Mycobacterium tuberculosis*	12.7 [8]	3.0
Influenza virus	100.0 [17]	25.0
Measles virus	570.0 [17]	143.0
Hypothetical	2000.0	500.0

**Table 2:** Waiting room default characteristics

Characteristic	Default value	Standard deviation
Length of room	8.00 m	n.a.
Width of room	6.00 m	n.a.
Height of room	2.75 m	n.a.
Volume of room	132.00 m^3^	n.a.
Room ventilation rate	4.0 Air changes per hour (outside air)	n.a.
Mean number of susceptibles	19	10
Mean pulmonary ventilation rate	0.48 m^3^/h [25]	0.20 m^3^/h [25]
Mean waiting time	30 minutes	10 minutes

n.a. = not applicable

Having determined the risk of airborne transmission under the default conditions, a sensitivity analysis was undertaken to determine the impact of variations in: (i) the room ventilation rate; and (ii) the mean waiting time, on the spread of disease.

## Results

**Figure 1:**
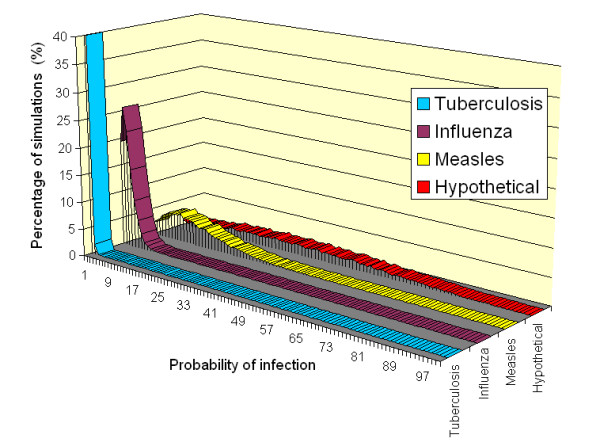
**Probability frequency distributions for the various diseases under the default conditions**.

Figure 1 shows the results of the Monte Carlo analysis for the four infections under the default conditions. From this it can be seen that, unlike the deterministic approach which yields a single probability value, the stochastic model returns a range of outcomes - from 'low risk' events in which the probability of infection is small, to 'high risk' events in which the chance of contracting an infection is much greater. From Figure 1 it is evident that as the quanta production rate increases so the frequency distribution curve lengthens and flattens, with the mean probability becoming greater and the number of 'high risk' transmission events greatly increasing. Consequently, susceptible individuals are at much greater risk of contracting an infection when quanta production rates are higher.

From Figure 1 above it can be seen that, as the quanta production rate rises, so the number of 'high risk' transmission events also increases. This phenomenon is particularly important, as it is these 'high risk' events that are most likely to result in the transmission of infection. Table 3 summarizes the probability frequency distribution curves shown in Figure 1 and gives a breakdown of the percentage of individuals at risk of contracting an infection for a range of quanta production levels. From this it can be seen that as the quanta production rate increases, so the percentage of simulations in which individuals have, say, a >10% risk of contracting an infection rises asymptotically. So while only 0.3% of influenza simulations result in a >10% risk, 59.1% of measles simulations result in the same risk of transmission.

**Table 3:** Frequency distribution of the probability of transmission in the waiting room under default conditions (assuming mean values for *p*, *t *and *N *of 0.48 m^3^/h, 30 minutes and 4 AC/h, respectively).

Infection	Mean quanta production rate (quanta/h)	Simulations resulting in a risk >1% [%]	Simulations resulting in a risk >5% [%]	Simulations resulting in a risk >10% [%]	Simulations resulting in a risk >20% [%]	Simulations resulting in a risk >30% [%]
TB	12.7	1.8	0.0	0.0	0.0	0.0
Influenza	100	81.4	10.1	0.3	0.0	0.0
Measles	570	97.6	83.3	59.1	21.0	5.4
Hypothetical	2000	99.4	96.5	91.6	78.5	61.2

**Figure 2:**
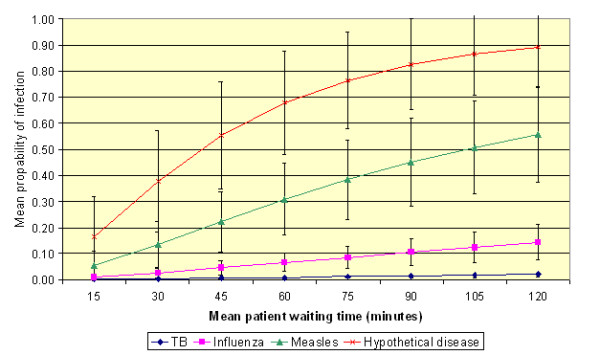
**The effect of varying patient waiting time on the risk of transmission under the default conditions (assuming mean values for *p *and *N *of 0.48 m**^**3**^**/h and 4 AC/h, respectively)**. (Error bars represent one standard deviation from the mean.)

**Figure 3:**
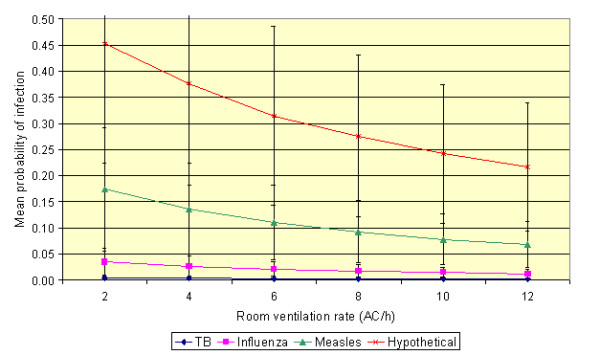
**The effect of varying patient waiting time on the risk of transmission under the default conditions (assuming mean values for *p *and *t *of 0.48 m**^**3**^**/h and 30 minutes, respectively)**. (Error bars represent one standard deviation from the mean.)

The results of the sensitivity analysis are presented in Figures 2 and 3. The results of varying patient waiting times are presented in Figure 2, which shows the mean probabilities for the four study infections. From this it can be seen that:

• The longer the patients spend in the presence of the infectious individual (i.e. waiting time), the greater the risk of transmission of infection.

• The greater the quanta production rate, the more non-linear the relationship between waiting time and the probability of infection.

• The risk of contracting TB or influenza is relatively small. However, the risks associated with the other two infections are much greater. Indeed, patients in the presence of an index case of measles or the hypothetical infection may become infected after relatively short exposure time.

For example, it can be seen from Figure 2 that the mean probability of acquiring a TB infection during a 30-minute stay in the waiting area is negligible (i.e. 0.0034), while that for influenza is an order of magnitude higher at 0.0262. By comparison the mean probability of acquiring a measles infection during the same period is 0.1349. If however, the duration of the stay is increased to 60 minutes then these values increase to 0.0087, 0.0662 and 0.3094, respectively.

The results of varying the room ventilation rate are presented in Figure 3, which shows the mean probabilities for the four infections studied. From this it can be seen that while increased ventilation rates reduce the risk of transmission for all the infections, at high quanta production rates there is still a relatively high probability that infection will spread even at a ventilation rate of 12 air changes per hour.

## Discussion and conclusion

The transmission of airborne disease is essentially a stochastic process, strongly influenced by chance events and system variance. It is therefore important to take this into account when modelling the airborne transmission of infection. The Gammaitoni-Nucci and Wells-Riley equations have frequently been applied in a deterministic manner [5,6,8]. Although this approach has merit, with a deterministic methodology it is only possible to determine the mean probability of a susceptible person becoming infected - it is not possible to predict those chance 'high risk' events that are most likely to result in the transmission of infection. Given that it is the 'high risk' events, rather than the mean probability that are critical, we adopted a stochastic methodology in our study to overcome some of the shortcomings associated with the deterministic approach.

From Table 3 it can be seen that as the quanta production rate rises, so the standard deviation of the results greatly increases. Consequently, the number of 'high risk' events associated with infections such as measles, are much greater than, say, for TB. Indeed, our analysis suggests that under normal conditions (i.e. without other complicating factors which might increase the production of droplet nuclei [5]) the chances of contracting tuberculosis in the waiting room under the default conditions are minimal. By comparison, if an infectious individual with measles is present in the waiting room, then under the same default conditions, the risk of transmission will be >10% on 59% of occasions, with the result that transmission of this disease is very likely to occur. This is reflected in the differences in observed attack rates for viral infections such as measles [6] and influenza [18], compared with those for open pulmonary TB. Indeed, our finding reinforces current guidance on TB prevention in the United Kingdom which recommends that periods of exposure to an infectious person in a confined space of less than eight hours should not be considered a significant risk [19].

Beggs *et al*. [5] demonstrated that length of exposure time plays a critical role in the transmission of TB. In most situations (i.e. situations where artificial aerosols are not generated) TB is not easily transmitted and long periods of exposure are generally required in order to contract an infection [2,5]. In the 1950s, Riley *et al*. undertook a study in a well-ventilated TB ward in a Baltimore, MD Hospital, from which they estimated that 'one unit of infection' was suspended in every 15 000-20 000 ft^3 ^(500-667 m^3^) of air in the ward [2]. From this they concluded that airborne *M. tuberculosis *bacilli were "not very numerous even in the vicinity of patients", and that it would take the "better part of a year" for a nurse working on a TB ward to breathe in 500-667 m^3 ^of air. The results of our study concur with this opinion and suggest that, although sporadic transmission may occur, the duration spent by most patients in waiting is too short for the risk of contracting TB to be anything other than minimal. Having said this, it should be noted that our study assumed: (i) the presence of only one infectious individual and (ii) a waiting area typical of that found in a UK hospital (i.e. not overcrowded). In many developing countries these assumptions may not be valid. In such countries hospital waiting areas are often very overcrowded, waiting times may be long (i.e. several hours), and more than one infectious individual might be present. Under such circumstances the risk of TB transmission is likely to be greater.

**Figure 4:**
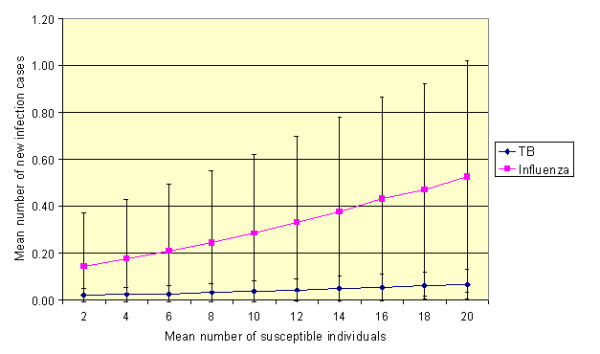
**The effect of varying the number of mean susceptible individuals within the waiting room under the default conditions (assuming mean values for *p*, *t *and *N *of 0.48 m**^**3**^**/h, 30 minutes and 4 AC/h, respectively)**. (Error bars represent one standard deviation from the mean.)

One factor that can vary greatly from day-to-day is the number of susceptible patients present in a waiting room at any given time. This, fact (often overlooked) is important because it has a profound effect on the number of people who are likely to contract an infection. If for example, the personal risk of contracting a given infection is constant, then equation 6 indicates that the likelihood of a person within the waiting area becoming infected is ten times greater if there are 20 susceptible people in the room, rather than just two. Although, the personal risk of contracting an infection is independent of the occupancy density (i.e. the probability of transmission is the same for each person in the waiting room), the fact that more people are present means that the chance that someone will become infected is much greater. In our model we allowed for this by varying the number of susceptible individuals present in each simulation. Figure 4 presents results of analysis undertaken for TB and influenza under the default conditions, assuming that the mean number of susceptible individuals varies. From this it can be seen, that for both infections, as the number of patients present increases, so the number of new infections occurring also increases, despite the fact that the personal risk to each individual in the space is nominally constant. Furthermore, it can be seen from Figure 4 that the standard deviation increases as the number of susceptible individuals present increases. Consequently, 'high risk' transmission events are more likely to occur when many individuals are present.

From the analysis above it becomes clear that the increased number of infection cases experienced in overcrowded spaces is simply a reflection of the fact that there are more susceptible individuals present to become infected. However, while this is undoubtedly true in part, in reality the whole picture is more complex, as proximity to an index case also influences personal risk. For example, if a person walks into a room containing one individual with open pulmonary tuberculosis, according to the Gammaitoni-Nucci model their personal risk would be the same whether there were six uninfected people in the room or 60. If, however, as a result of having 60 people in the room space an individual was forced into close proximity with the index case, then their risk of contracting an infection would probably increase. However, because of the logistical and computational difficulties associated with simulating proximity, it was not possible in our study to assess this issue.

In order to prevent the transmission of airborne infection many researchers have advocated the use of improved building ventilation [8,12,20] or air disinfection devices [21-23]. While such strategies undoubtedly have potential in applications where exposure times are long, their use in the waiting areas of healthcare facilities appears to be much more questionable. Despite this ultraviolet lamps were reported to be installed in 12/144 (8%) and HEPA (high efficiency particulate air) filtered air provided in 22/138 (16%) of Emergency Department waiting areas in facilities which recorded >1 case of TB per month [24]. From Figure 3 it can be seen that little impact is made on the transmission of TB and influenza by increasing the ventilation rate to 12 air changes per hour. This is because the exposure times are generally too short for these infections to have much impact. Any benefit derived from increased ventilation is significantly outweighed by factors such as the exposure time and the numbers of susceptible individuals present. Even with high quanta producing diseases, such as measles, where improved ventilation might be beneficial, reducing: (i) waiting times; and (ii) the number of susceptible individuals present, appear to be as important as installing expensive ventilation/air cleaning equipment. It is therefore important when seeking to prevent the transmission of airborne viral disease to first minimize waiting times and the number of susceptible individuals present before turning to expensive technological solutions.

While the Gammaitoni-Nucci model calculates the risk that an airborne disease might be transmitted in a confined space, it is important to remember that the results it produces rely wholly on the quality of the data used. With respect to this, the room volume, room ventilation rate, average pulmonary ventilation rate, and average occupancy time, are all variables that can be estimated with some degree of accuracy. However by comparison, the quanta production rate is much harder to estimate. This is because there is a paucity of good quality data regarding quanta production rates. Values are generally calculated retrospectively, using either the Gammaitoni-Nucci or Wells-Riley equations after an outbreak has occurred. Furthermore, published quanta values can vary greatly for the same disease, making comparisons difficult. For example, while Riley *et al. *[4] estimated that the average TB patient in hospital produced only 1.25 quanta per hour, Nardell *et al. *[8], investigating a TB outbreak in a Massachusetts office building, calculated the infectious dose to be 12.7 quanta per hour. Given the variability in the published quanta production data, the results generated by the Gammaitoni-Nucci model should only be considered as indicative of trends rather than absolute values. Furthermore, because infectious individuals are likely to generate differing quanta production rates, it is important when modelling the risk of transmission to use a range of values, rather than use a single fixed quanta production rate.

## Competing interests

The authors declare that they have no competing interests.

## Authors' contributions

CBB and SJS designed the study. CBB developed the computer model and KGK advised on the clinical aspects of the study. CBB wrote the manuscript with major contributions from other authors. All authors have read and approved the final manuscript.

## Pre-publication history

The pre-publication history for this paper can be accessed here:

http://www.biomedcentral.com/1471-2334/10/247/prepub
